# A variant of the gene *HARS* detected in the clinical exome: etiology of a peripheral neuropathy undiagnosed for 20 years

**DOI:** 10.1515/almed-2020-0033

**Published:** 2020-05-19

**Authors:** Raquel Lahoz Alonso, Paula Sienes Bailo, Jose Luis Capablo Liesa, Sara Álvarez de Andrés, Jose Luis Bancalero Flores, Silvia Izquierdo Álvarez

**Affiliations:** Department of Clinical Biochemistry, Hospital Universitario Miguel Servet, Zaragoza, Spain; Department of Neurology, Hospital Universitario Miguel Servet, Zaragoza, Spain; NIMGenetics, Madrid, Spain

**Keywords:** Charcot-Marie-Tooth disease, axonal, type 2W, exome, HARS protein, human

## Abstract

**Objectives:**

Describe a case with axonal Charcot-Marie-Tooth (CMT) type 2W, a neurological disease characterized by peripheral neuropathy typically involving the lower limbs and causing gait alterations and distal sensory-motor impairment.

**Case presentation:**

We report this case, where the application of massive genetic sequencing (NGS) with clinical exome in a molecular genetics laboratory enabled to detect the presence of candidate variants of the clinic of the patient.

**Conclusions:**

The variant detected in *HARS* gene suggests that this variant could be causative of the symptoms of the patient, who went undiagnosed for 20 years and experienced an exacerbation of symptoms over time.

## Introduction

Charcot–Marie–Tooth (CMT) neuropathy encompasses a heterogeneous group of hereditary diseases affecting peripheral nerves. With a prevalence of one per 2500, the onset of symptoms typically occurs in adolescence or early adulthood in the form of progressive muscle weakness and limb atrophy, sensory loss, skeletal deformities, gait impairment, and loss of reflexes, although the intensity of symptoms varies even among patients with the same genetic mutation [1].

In neurophysiological terms, CMT is classified into three types: CMT1 is prevailingly demyelinating, with slowed nerve-conduction velocities (CV) of ≤38 m/s in the upper limbs. CMT2 is prevailingly axonal, with preserved CVs but reduced muscle action potential amplitudes secondary to axonal degeneration. Intermediate CMT has demyelinating and axonal characteristics, with CVs ranging from 25 to 45 m/s. A curative therapy has not yet been developed for CMT and the management of patients involves physical and occupational therapy, splint immobilization, use of orthopedic devices, and orthopedic surgery to address the disabling symptoms of the disease [2].

CMT is transmitted as a recessive or X-linked autosomal-dominant trait [3]. To date, more than 80 genes have been associated with CMT [4]. In CMT2, mutations in the mitofusin 2 gene (*MNF*2) are associated with the most common subtype of CMT2A, accounting for 10–30% of mutations [5]. However, the causative gene of the disease cannot be identified in a third of cases as a result of genetic heterogeneity, pleiotropy, and absence of other genes responsible for a large proportion of cases [3, 6, 7].

Five of the genes associated with CMT encode different aminoacyl-tRNA synthetases (ARSs), which are the enzymes responsible for charging tRNAs molecules with amino acids that are transferred onto a growing peptide during ribosome translation: glycyl-(GARS) ***,*** tyrosyl-(YARS), alanyl-(AARS), tryptophanyl-(WARS) and histidyl-tRNA synthetase (HARS) [8], [9], [10], [11], [12]. With regard to *HAR*SHARS, previous studies have documented a relationship between mutations in this gene and CMT [13]. More recent studies assessing the physiopathology of the disease have revealed a relationship between *HAR*S mutations and a reduced catalytic activity of the HARS enzyme [14, 15].

## Case presentation

We report the case of a 34 year-old man referred from the Department of Clinical Genetics of Hospital Universitario Miguel Servet (HUMS) located in Zaragoza, Spain, with motor axonal polyneuropathy with mild or uncertain sensory loss.

At 8 years of age, the patient exhibited elevated levels of total immunoglobin E (IgE, 560 UI/mL) that persisted over the years, with a peak of 2484 UI/mL. Allergies and intestinal parasite infection were ruled out. At age 23, the patient had developed a progressive peripheral neuropathy that involved the lower limbs.

Neurophysiological studies revealed the presence of severe muscle denervation and motor axonal neuropathy in the lower limbs, with very-dominant, bilateral, symmetric distal involvement. Sensory studies were normal in the upper limbs and demonstrated trivial alterations in the lower limbs. From the age of 14, the patient exhibited pes cavus and hammer toes with difficulty in running and bouncing, and foot and leg pain. Family history was unremarkable, except for his mother, who had tremors.

Upon suspicion of an inherited peripheral neuropathy (CMT type), next generation sequencing (NGS) of a panel of 34 CMT-associated genes was performed (Sistemas Genómicos, ASCIRES, Valencia, Spain) (Supplementary Material, Annex 1). None of the pathogenic variants linked to CMT was detected in the panel of genes analyzed, only a variant of unknown significance (VUS) in the *KARS* gene (Table 1), according to American College of Medical Genetics and Genomics (ACMG) standards and guidelines for the interpretation of sequence variants (Genetics in Medicine, 2015). This variant is not described in the Human Gene Mutation Database (HGMD) and Leiden Open Variation Database (LOVD) databases and is identified with a recessive allele frequency of 0.1% in the dbSNP database. In addition, *in silico* studies do not show a clear splicing alteration secondary to the presence of this variant, which has been recently rated as benign by several authors in the ClinVar database. However, in view of the lack of correlation with the phenotype of the patient and the recessive nature of the disease with which the mutations of this gene are associated, even although the pathogenicity of this variant was confirmed, the symptoms of the patient can only be explained by the presence of another alteration.

**Table 1:** Genetic studies performed in the patient and his parents.

	Gene	Variant nomenclature	Exon	Cygosity	Effect	Variant categorization	Inheritance	Phenotype
A	*KARS*	c.696A>G p.(Thr232Thr)	6	Het	UD	VUS	AR	Intermediate type B CMT
B	*HARS*	c.397G>T p.(Val133Phe)	5	Het	*missense*	VUS	AD	Axonal CMT type 2W
C	*MUT* *YH*	c.1187G>A p.(Gly396Asp)	13	Het	*missense*	PV	AR	Family adenomatous polyposis
D	*HARS*	c.397G>T p.(Val133Phe)	5	Het	*missense*	VUS	AD	Axonal CMT type 2W
E	*MUT* *YH*	c.1187G>A p.(Gly396Asp)	13	Hom	*missense*	PV	AR	Family adenomatous polyposis

Het, heterozygosis; Hom, homozygosis; UD, undetermined; VUS, variant of unknown significance; PV, pathogenic variant; AR, autosomal recessive; AD, autosomal dominant. Genomic variants identified in: (A) panel of 34 genes assocaited with CMT in the patient (Sistemas Genómicos, ASCIRES, Valencia, Spain) and (B) clinical exome in peripheral blood (ExoNIM^®^ NIMGenetics). Incidental finding in the clinical exome of the patient according to *American College of Medical Genetics and Genomics* guidelines (ACMG) (C). Evaluation of the pattern of segreation of the variants identified in the patient by Sanger sequencing: (E) in the father (NIMGenetics, Madrid, Spain) and (D) in the mother (Cerba Internacional, Sabadell, Barcelona).

New electroneurographic and electromyographic studies confirmed suspicion of axonal neuropathy and demonstrated severe motor neuropathy that only affected the lower limbs. In view that conduction velocities and distal latencies were normal, demyelinating neuropathy was ruled out. However, a neurogenic pattern with intense signs of denervation and loss of motor units was identified in the associated musculature. In sensory nerves, amplitudes were in the lower limit of normal.

**Figure 1:**
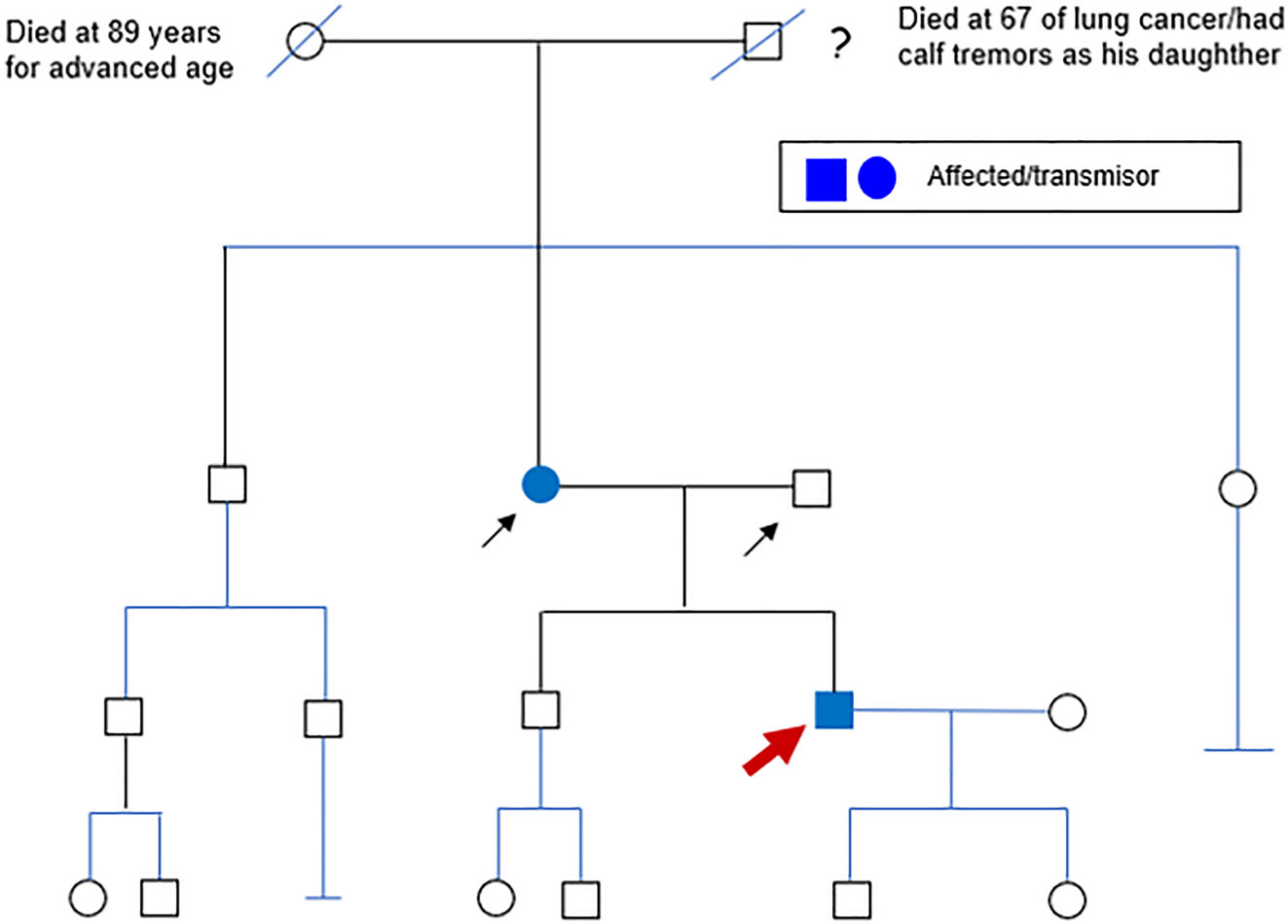
Pedigree. The arrows point out the family members studied.

NGS was complemented with clinical exome sequencing (ExoNIM^®^ NIMGenetics, Madrid, Spain). As a result, the heterozygous variant described in Table 1 was identified. This variant is described in the ClinVar database based on evidence from a single case report (Variation ID: 548118) as a probably-pathogenic variant associated with axonal CMT type 2W (MIM#616625). In the same line, pathogenic missense variants associated with peripheral neuropathy are described in adjacent codons in the HGMD database. As an incidental finding, we identified a pathogenic variant of the *MUTYH* associated with autosomal recessive familial adenomatous polyposis (Table 1). The segregation pattern of the *HARS* c.397G>T; p.(Val133Phe) and *MUTYH* c.1187G>A; p.(Gly396Asp) variants was analyzed in his parents by Sanger sequencing to investigate whether they were *de novo* or inherited variants (Figure 1).

The father was found to be a homozygous carrier of the *MUT* *YH* c.1187G>A; p.(Gly396Asp) variant, which confirmed the paternal inheritance of this variant (Table 1) (NIMGenetics, Madrid, Spain). The mother, who exhibited electromyographic alterations, was a heterozygous carrier of the c.397G>T; p.(Val133Phe) variant, which confirmed the maternal inheritance of this variant (Table 1) (Cerba Internacional, Sabadell, Barcelona, Spain). Unfortunately, the presence of these variants could not be assessed in other family members.

**Figure 2:**
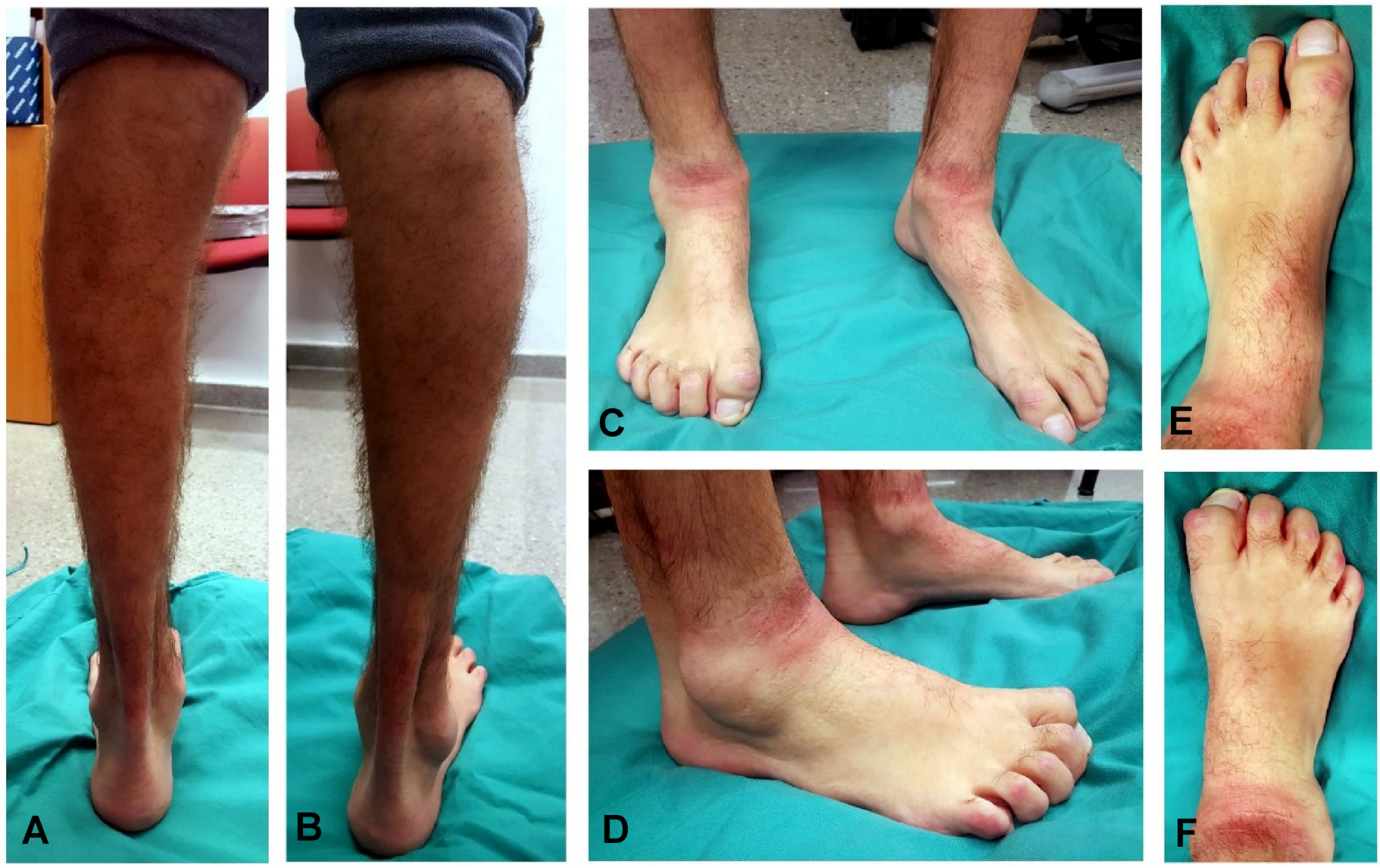
Clinical phenotype of the patient. Calf atrophy in the (A) left and (B) right leg. (C-D) show frontal and lateral images of the feet. The left (E) image shows a slight dorsal flexion. The right (F) shows no dorsal flexion, the first toe is rigid, with a dorsal callus. The second is also almost rigid.

At present, the patient has a neurological foot, with hammer toes and calf atrophy, and describes fatigue and weakness in the lower limbs (Figure 2). The patient is currently on curcumin and diet therapy, with fasting periods. This treatment has been proven to increase the number and size of myelinated axons and improve motor performance in Tembler-J mice [16]. In addition, the patient uses shoe pads and follows an individualized exercise program.

## Discussion

The presence of variants of the *HARS* gene is associated with axonal CMT type 2W (MIM#616625), with an autosomal dominant inheritance pattern. CMT type 2W is a neurological disease characterized by peripheral neuropathy typically involving the lower limbs and causing gait impairment and distal sensory loss. However, most patients also develop upper limb problems as the disease progresses. In this case, the absence of sensory loss and upper-limb symptoms may be due to the young age of the patient, as distal sensory alterations generally appear over the years or with pure motor neuropathy, which is considered a different phenotype of this disease.

The heterozygous Val133Phe variant identified in this patient is a missense variant that is described in the ClinVar database as a probably pathogenic variant based on evidence from a single study. This mutation was first reported by Royer–Bertrand et al. in 2019 [14], who documented a deleterious effect of this variant on HARS function, as it affects highly-conserved amino acids located in the catalytic domain, although the expression of these proteins in cells is not altered.

In the same vein, pathogenic missense variants associated with peripheral neuropathy are described in adjacent codons ([c.395C>T p.(Thr132Ile), c.401C>A p.(Pro134His)]) in HGMD, ClinVar and UniprotKB databases [12, 13], which supports the hypothesis of a potential relationship between this variant and the symptoms exhibited by our patient.

**Figure 3:**
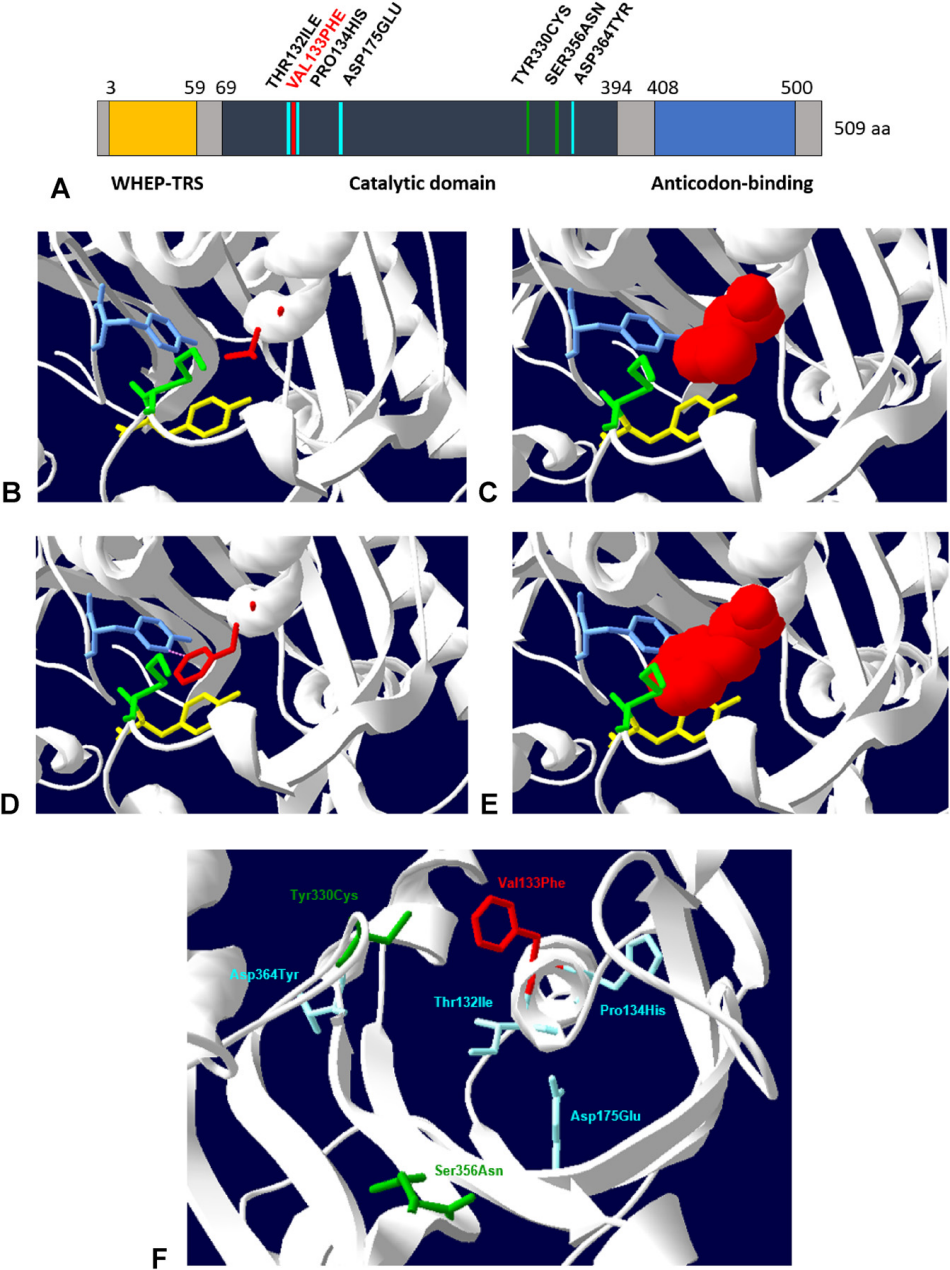
Domains of histidyl-tRNA synthetase (HARS). (A) Domains of HARS. The variants described in the literature have been included. V133F substitution described in this report is in red. (B–E) Location of these variants associated with CMT in the active HARS site (PDB 4PHC). The different substitutions appear in a 3D structure on the representation of the secondary structure of the protein. (B–E) show the steric hindrance that appears in residue 133 of the catalytic core when the protein exhibits V133F substitution (Phe133, images D–E) vs when it is in its native form (Val133, images B–C). (B) and (D) shows 3D representations of the variant V133F (red) and amino acids of the catalytic core with which it interacts (yellow-Tyr107, greed-Lys106 and blue-Tyr330). (C) and (E) include the representation of Van der Waals radii in 3D surfaces. (F) displays the other mutations described in the literature (mutations described by Brozkova et al. are in blue, whereas those described by Abbott et al. are in green) [12, 13]. (B, F) were constructed using SPDBviewer.

Bioinformatic analysis predicts that this variant compromises the structure or function of the protein encoded by the *HAR*S gene. 3-D modeling programs suggest that substitution of Val133Phe with Lys106, Tyr107 and Tyr330 results in steric hindrance (Figure 3). The Human Splicing Finder software predicts that this variant causes aberrant splicing, which compromises mRNA transit.

When analysis of the mutations typically associated with CMT does not explain clinical symptoms in a patient, NGS and clinical exome sequencing will yield rare variants or variants - either alone or in combination with other variants - not previously known to cause patient’s phenotype, or lead to incidental findings. In this case, clinical exome sequencing enabled us to identify a heterozygous Gly396Asp mutation in the *MUTHY* gene, which indicates a predisposition to colon cancer. In view of this finding, exome sequencing was performed in his parents, which led to early diagnosis of CMT2W in the mother and family adenomatous polypomatosis in the father. The fact that CMT2W had not been diagnosed in the mother earlier may be explained by incomplete penetrance of the allele and variability in the clinical manifestations of the disease in a family. With regard to the father, the identification of family adenomatous polypomatosis enabled adequate genetic counseling and the adoption of preventive measures.

Lessons learned

The presence of *HARS* variants is associated with axonal CMT type 2W (MIM#616625), with an autosomal dominant inheritance pattern.
Axonal CMT type 2W is a neurological disease characterized by peripheral neuropathy typically involving the lower limbs and causing gait impairment and distal sensory loss.
The use of massive sequencing techniques (i. e. NGS) has facilitated significantly the identification of causative mutations of hereditary neuropathies such as CMT.
Clinical exome sequencing is a useful tool that enables mapping from the genotype to the phenotype in patients with clinical symptoms of unknown etiology.
Massive sequencing techniques and clinical exomes occasionally lead to incidental findings that facilitated the provision of genetic counseling to the patient and his family.

## Supplementary Material

Supplementary Material Details
