# Supplementary material for: A variant of the gene HARS detected in the clinical exome: etiology of a peripheral neuropathy undiagnosed for 20 years
Source: Adv Lab Med. 2020 May 19;1(4):20200033. doi: 10.1515/almed-2020-0033 (PMC10197443; doi:10.1515/almed-2020-0033)
Supplement: Supplementary file 1 — Supplementary Material Details [file j_almed-2020-0033_suppl.docx]

**Annex 1. NGS panel of 34 genes associated with Charcot-Marie-Tooth diseaes (Sistemas Genómicos, ASCIRES, Valencia, Spain).**

Genes studied: *AARS, ARHGEF10, DNM2, DYNC1H1, EGR2, FGD4, FIG4, GARS, GDAP1, GDAP1L1, GJB1, HK1, HSPB1, HSPB8, KARS, KIF1B, LITAF, LMNA, LRSAM1, MED25, MFN2, MPZ, MTMR2, NDRG1, NEFL, PRX, PMP22, PRPS1, RAB7A, SBF2, SH3TC2, SPTLC1, TRPV4, YARS*.
